# Lessons from nature: achieving postural balance inspired by biomechanics and natural (a)symmetry. A perspective article

**DOI:** 10.3389/fpsyg.2026.1736186

**Published:** 2026-07-13

**Authors:** Alexandra Türk-Espitalier

**Affiliations:** Center of Music Physiology, University of Music and Performing Arts Vienna, Vienna, Austria

**Keywords:** asymmetry, instrument, knowledge transfer, music pedagogy, nature, playing posture, symmetry

## Abstract

**Introduction:**

Musicians playing asymmetrically held instruments are particularly susceptible to playing-related musculoskeletal disorders (PRMD). But a sustained asymmetrical posture is unavoidable, making it essential for musicians to manage the demands that this asymmetry places on their body. Standard PRMD prevention includes education on physical competence, healthy practice habits, and tailored exercises. However, the author hypothesizes that musicians could also benefit from a nature-inspired approach alongside established preventive measures.

**Aim:**

This perspective article aims to present how natural patterns of balance and harmony seen in animal movement and plant structures can be transferred and embodied into ergonomic approaches and postural adjustments for musicians.

**Methods:**

The perspective emphasizes the pedagogical process of transferring knowledge from nature to music. To translate principles from physics, biomechanics, biology, and design from the natural world to instrumental posture, established learning concepts—using lectures, self-study and study groups—can be applied. The process begins with factual knowledge about nature, followed by conceptual knowledge, where similarities between principles in nature and music are discovered. Procedural knowledge then transforms the “what” into “how”, using images, visualization, and body awareness exercises to embody new movements and ideas. Finally, metacognitive knowledge involves self-reflection and critical awareness about the implementation process and its outcomes.

**Conclusion:**

This proposal should be seen as an inspiration for musicians playing asymmetrically held instruments to open their minds to a world where symmetry and asymmetry coexist seamlessly. Nature can serve as a source of such inspiration, offering countless examples in which form adapts flexibly to functional demands.

## Introduction

A musician's life offers artistic fulfillment but is also associated with considerable physical and psychological demands. Among the most prevalent musicians' diseases are playing-related musculoskeletal disorders (PRMD) (Steinmetz et al., 2015; Rotter et al., 2020; Rodríguez-Gude et al., 2023). PRMD are defined as “any pain, weakness, numbness, tingling, or other physical symptoms that interfere with your ability to play your instrument at the level you are accustomed” (Zaza and Farewell, 1997; Zaza, 1998). These disorders can affect muscles, tendons, joints, and nerves, most commonly in the neck, shoulders, back, and upper extremities (Spahn et al., 2011).

While prevalence estimates vary depending on study design (Spahn et al., 2011; Rotter et al., 2020), rates of PRMD can reach up to 89% in orchestra musicians (Steinmetz et al., 2015; Kok et al., 2016) and 76% in adolescent musicians (Gembris et al., 2020). Although no consensus has yet been reached regarding the exact causality and etiology of PRMD (Baadjou et al., 2016), several contributing factors have been identified, including repetitive movements, awkward playing postures, prolonged practice hours, faulty technique, and postural stress inherent to instrumental performance, all of which may contribute to muscular overload, imbalance and fatigue (Ackermann and Driscoll, 2010; Gembris et al., 2018; Nusseck and Spahn, 2020; Shinde and Borkar, 2021; Ballenberger et al., 2023).

The specific physical challenges differ between instruments. For example, violinists and violists must support the instrument between the left shoulder and chin while performing asymmetrical arm movements, flutists typically maintain a rotated upper-body posture. These musicians, who play asymmetrically held instruments show significantly higher rates of PRMD and greater postural asymmetries than those with a symmetrical playing posture (Wahlström Edling and Fjellman-Wiklund, 2009; Ackermann et al., 2012; Barczyk-Pawelec et al., 2012; Steinmetz et al., 2012; Rensing et al., 2018). But as a professional musician playing an asymmetrically held instrument, this sustained asymmetrical posture is unavoidable. It is therefore essential that musicians develop strategies to cope with the physical challenges resulting from this asymmetry. Standard approaches to prevent PRMD focus on health education, the development of healthy practice routines, and tailored exercise programs, including physiotherapy, posture training, and strength and flexibility exercises (Barton and Feinberg, 2008; Chan et al., 2013; Spahn et al., 2015; Spahn, 2017; Matei and Phillips, 2023).

While exercises and targeted interventions can help reduce PRMD (Chan and Ackermann, 2014; Chan et al., 2014; Roos et al., 2024), the content of these recognized methods is primarily derived from medicine, anatomy, and exercise science. But given that PRMD is a multifactorial problem, it may be beneficial to add a further component to conventional preventive measures—one that broadens the perspective on managing PRMD and asymmetric playing posture.

The author hypothesizes that nature could fulfill this role. With its structural patterns of balance and harmony, plants and animals not only provide artistic inspiration but may also promote ergonomic strategies, fluent movements, and healthy posture.

This perspective article aims to present a theoretical concept of how nature can support musicians in addressing a situation that cannot be changed—namely, the asymmetric playing position. The concept combines two complementary approaches: First, it utilizes examples from nature to improve musicians' understanding of their own body, movement patterns, and postural organization, all with focus on (a)symmetry. Second, it uses nature as a source of knowledge and artistic inspiration, encouraging musicians to explore alternative perspectives on (a)symmetry, balance, and movement that may enrich both their physical performance and artistic expression. Both approaches seek to inspire musicians to open their minds to unconventional thoughts and use these insights to amplify positive effects of established methods.

### Symmetry and asymmetry in nature and the human body

#### Biological background

An object or organism is considered symmetrical if it remains unchanged in appearance after a geometric transformation (Hudson, 2000; Benítez et al., 2020). This can be a reflection along a plane, a shift or a rotation (Hudson, 2000). Three types of symmetry exist in organisms. Most animals that show directed locomotion are externally bilaterally symmetrical. Prominent examples are butterflies or the human skeleton. Here, a right and left side is evident and if mirrored along the sagittal plane, both parts are congruent (Benítez et al., 2020; Mainzer, 2022). Radial symmetry is prevalent in flowers, and in animals that don't move much, such as starfish or sea anemones (Haeckel, 2004; Benítez et al., 2020). They do not feature a right or left side, but a lower and upper one instead (Benítez et al., 2020; Mainzer, 2022). Approximate spherical symmetry with unlimited axes of rotational and mirror symmetry is observed in certain viruses such as the SARS-CoV-2 and in microalgae (Encyclopaedia Britannica, n.d.; Hudson, 2000). The form of symmetry in animals arises from functional demands that ensure their survival (Mainzer, 2022). Radial symmetry is advantageous in aquatic environments, where it facilitates floating with ocean currents, whereas bilateral symmetry enables directed swimming as well as efficient locomotion on land and in the air (Mainzer, 2022). Thus, symmetry can be regarded as functionally beneficial.

However, it is also fragile and often broken (Benítez et al., 2020; Mainzer, 2022; Bersuker, 2023). Symmetry breaking is observed across multiple scales in nature, from elementary particles to biological systems (Muñoz-Nortes et al., 2014; Mainzer, 2022; Bersuker, 2023). Genetic and environmental stress can cause these small, random deviations from perfect bilateral symmetry, leading to a state called fluctuating asymmetry (Tomkins and Kotiaho, 2002; Graham et al., 2010; Benítez et al., 2020). Fluctuating asymmetry can be easily witnessed externally in animals and plants. Trees, for example, grow in a naturally balanced form when standing solitary without competition. But environmental conditions are rarely ideal, so they must adapt to light (phototropism), wind and mechanical stress (anemotropism and thigmomorphogenesis), gravity (gravitropism), water (hydrotropism and xerophytism) and temperature (thermotropism) during growth (Chehab et al., 2008; Harmer and Brooks, 2018; van Zanten et al., 2021; Gul et al., 2023). As a result, trees may develop markedly asymmetric growth forms, such as wind-shaped canopies, leaning trunks, or one-sided crown development.

#### Aesthetic perception

Numerous examples in both flora and fauna demonstrate that symmetry and asymmetry coexist in natural processes (Sabelli et al., 2010; Damerval et al., 2021). Hereby, symmetry is considered to represent repetition and therefore stability, whereas asymmetry brings disorder and instability (Hudson, 2000; Polak, 2003; Benítez et al., 2020). Because of its order, symmetry is generally labeled as beautiful (Hudson, 2000; Sabelli et al., 2010) and asymmetry as ugly (Lin, 2001). However, symmetrical uniformity can also be boring and asymmetrical complexity interesting, thus making the monotonous symmetry ugly and the multifaceted asymmetry beautiful (Lin, 2001; Sabelli et al., 2010; Lüttge and Souza, 2019). Highly symmetrical human faces are perceived as beautiful, but in order to not appear artificial and life-less they need a slight asymmetry to add charm, dynamic and emotion (Sabelli et al., 2010; Lüttge and Souza, 2019).

Beauty and harmony are also evident in forms beyond radial and bilateral symmetry. From the structure of DNA to the travel paths of planets, harmonic patterns and principles of organization, such as waves and spirals and can be detected throughout nature. Notable examples include nautilus shells, pinecones, and sunflower seeds, whose structural arrangement follows the golden spiral (Lüttge and Souza, 2019). The golden ratio is likewise observed in the pentagram of five-petaled flowers (Sparavigna and Baldi, 2016). Although asymmetrical, the golden ratio and the golden spiral are considered exceptionally harmonious and beautiful. For musicians, these examples illustrate that asymmetry does not necessarily imply dysfunction, deficiency, problems, or pain. Understanding that balance, efficiency, and beauty can also emerge from asymmetrical structures may help musicians develop a more functional and less restrictive perception of their own playing posture.

#### Human body

Similar to nature, in the human body, bilateral symmetry, but also certain asymmetries and proportions approximating the golden ratio can be observed, without excluding one another (Sabelli et al., 2010; Thomas et al., 2020; Naini, 2024). The human musculoskeletal system is clearly bilaterally symmetrical; however, muscles and myofascial chains are also arranged in a spiral pattern. Furthermore, human and four-legged locomotion in most animals is diagonal, and functional and neuronal body laterality is represented by handedness and footedness as well as eye dominance. Most of our organs are also not paired or bilaterally symmetrical.

When playing an instrument, the left and right side of the body perform different tasks. However, in asymmetrically held instruments, not only do the hands and fingers move differently and independently of one another, but the fundamental whole-body posture also differs markedly between the left and right sides. But musicians are not static figures that have to rely solely on their bilateral symmetry to maintain posture (Mantel, 2011). Instead, they move and must find an overall postural and dynamic muscular balance together with their instrument, even when it is held exclusively to one side (Mantel, 2011; Türk-Espitalier, 2016). Recognizing that the human body is multifaceted—and that skills such as balance, movement within postural alignment, and body-awareness are essential processes and crucial for managing asymmetric tasks—can constitute the first step toward a more natural approach to playing an asymmetrically held instrument (De Alcantara, 2013).

### Transfer to the instrument

The overarching goal of this concept is to recognize, transfer, and implement situations of natural (a)symmetry into instrumental practice. But transfer processes can be challenging, especially when they involve a field seemingly far away from the one where a person is the expert. Structuring the learning contents along the four knowledge dimensions—factual knowledge, conceptual knowledge, procedural knowledge, and metacognitive knowledge—described in Bloom's taxonomy of learning can be an option (Bloom, 1956, 1971; Anderson and Krathwohl, 2001). Despite previous critical evaluations (Ormell, 1974; Krathwohl, 2002), this model provides clear guidance, and the author therefore considers it suitable for the concept.

For musicians, acquiring factual knowledge about human anatomy, biomechanics, the risk of PRMD, and symmetry and asymmetry in both instrumental posture and nature can serve as a foundational first step. The second step, conceptual knowledge, involves discovering and identifying parallels between principles in nature and in music. In small groups or individual practice sessions, students may analyze their posture, explain how asymmetry relates to their performance and compare it with similar situations in nature. For example, flutists may relate the rotational demands of their playing posture to the spiraling growth patterns observed in climbing plants, using this imagery to distribute rotational movements throughout the trunk rather than concentrating them in the neck or shoulders.

The next stage, procedural knowledge, involves mastering subject-specific techniques and methods, while understanding the conditions under which they should be applied. Procedural knowledge turns the “what” into the “how”, with imagery, visualization, and exercises facilitating the embodiment of new ideas at the instrument. Key elements of this stage are body awareness, balance, gravity, three-dimensionality, and the connection between sound and movement. Body awareness exercises stimulate proprioceptive functioning and can be executed with or without instrument (Türk-Espitalier, 2016). A heightened proprioception can lead to a better feeling of posture and alignment. This is crucial for musicians playing asymmetrically held instruments, as postural misalignment can be detected among those instrumentalists not only while playing but also in daily life (Nusseck and Spahn, 2020). A proper alignment forms the foundation for balance. Just as a tree with asymmetrical branches can remain stable through balanced growth, a flutist or violinist can learn to balance asymmetrical playing demands without unnecessary muscular fixation. For example, a violinist who habitually uses too much muscle tension in the left shoulder may use the image of a tree yielding to wind forces rather than resisting them through rigidity. In practical terms, musicians can learn exercises that promote movement from the body center, while the upper extremities perform different movements or assume varied positions, thus helping to perceive and maintain overall equilibrium. Additionally do subtle weight shifts between the feet and small rotational movements of the spine facilitate the prevention of a prolonged static posture and reduction of early muscular fatigue (Garcia et al., 2015).

Balance is closely linked to gravity and efficient force transmission. Regardless of the task—the center of mass must be aligned with the vertical axis. Also, large, weight-bearing joints such as the sacroiliac joints and hip joints should be well centered in order to transfer forces in a targeted and efficient way. Knowledge of anatomy and biomechanics can facilitate physical perception of joint alignment and muscular action during movement (Sohier, 1999). Furthermore, it can encourage musicians to think and feel in forces instead of form (Sohier, 1999; Enoka, 2015), which can lead to better management of action-reaction coupling and more fluent and controlled movements. The animal world offers numerous examples of good alignment, efficient movement, and adaptive responses to forces. For example, the cheetah's ability to combine speed with elasticity and efficiency may inspire violinists to perform shifts with fluidity and minimal unnecessary tension, rather than relying on rigid muscular power. Subsequently, this enhanced proprioceptive and neuromuscular control can decrease the risk of PRMD (Cardoso et al., 2025). Moving naturally balanced does not only have physical advantages for the musculoskeletal system, but also greatly enhances sound quality (Mantel, 2011; Türk-Espitalier, 2016). Posture and movement always happen in all three dimensions and no matter which instrument, musicians should seek for movements that offer space, freedom and openness, which will be reflected in equally free and fluent musical phrasing.

Suitable exercises for the embodiment process in the procedural knowledge stage can be drawn from established body-oriented approaches that support fluid, well-coordinated movements and promote a calm, focused mindset (Spahn, 2017). Functional strength training and stretching may counterbalance muscular asymmetry (van den Berg, 2011; Türk-Espitalier, 2016), and core and stance stabilization exercises support the body in coping with the unilateral lever forces produced by the arms and the instrument. Like an asymmetric tree crown being supported by an expanded root system, these exercises can help musicians counterbalance asymmetrical loading and maintain long-term postural stability.

The fourth step—metacognitive knowledge—is characterized by self-reflection and critical awareness of both the embodiment process and its outcomes. Musicians can ask themselves how asymmetry affects them musically and physically and whether the insights gained in the previous steps have changed their practice behavior and mindset. Furthermore, they can investigate what other concepts or mindsets could be beneficial (De Alcantara, 2013; Hinsdale and Ljungblad, 2023).

The staged progression through the four knowledge dimensions provides a structured framework for transferring nature-based insights into an applied understanding of asymmetrical playing posture and movement. Factual knowledge forms the foundation, while the actual transfer and embodiment primarily occur within the conceptual and procedural stages. Table 1 provides examples and exercises that facilitate this transfer. A final phase of critical reflection on the preceding steps completes the process, after which musicians will be able to identify examples of (a)symmetry in nature and music, explain the relationship between natural (a)symmetry and instrumental posture, demonstrate posture and movement strategies to counterbalance asymmetry in playing, reflect critically on their own playing posture, and develop personalized strategies for healthy performance.

**Table 1:** Toolbox example: using nature as a model for movement and artistic inspiration.

Purpose	Nature example	Application	Exercises/techniques
Spinal length	Straight, long tree	Decompression of the spine	Gyrotonic, Gyrokinesis
Balanced movement	Swaying tree	Reduce rigidity in posture	Ideokinesis, Tai Chi
Myofascical integration	Climbing plant spirals	Rotational coordination	Spiral dynamics, myofascial unwinding
Force transmission	Jumping cheetah	Enhance elasticity	Use of elastic resistance band or gymnastic ball
Artistic inspiration	Birdsong	Musical expression, phrasing	Transfer to adequate pieces, e.g. “Le merle noir” by O. Messiaen

## Discussion

Nature has always served as a profound source of inspiration for artists. Painters, poets, and composers have captured both its beauty and its cruelty in their works, while performers have drawn on nature to shape their interpretations. But besides its artistic inspiration, nature also provides models for technological innovation, as demonstrated in bionics. Furthermore, as suggested in this perspective article, it can offer analogs for human movement, including postural organization and movement strategies in an asymmetric playing position.

The perspective is intended as a conceptual framework rather than a validated pedagogical method. Its aim is to stimulate new perspectives on (a)symmetry, movement, and playing-related musculoskeletal disorders (PRMD). The two suggested approaches—one, using nature to understand bodily organization in relation to (a)symmetry, and the second using nature as a source of knowledge and artistic inspiration that broadens musicians' perspectives on movement and artistic expression—should both be applied and integrated into the daily practice routine. To facilitate dissemination among musicians, the concept can be organized into structured modules or practical workshops with high applicability, as demonstrated in Table 1.

The novelty of the proposed framework is not the use of imagery or body-awareness training itself, both of which are well established in music pedagogy. Rather, it lies in the systematic use of natural forms, growth processes, and adaptive strategies as observational models for exploring alternative movement solutions in instrumental practice. Viewing an asymmetrical playing position as a substantial cause of PRMD, and seeking to reduce PRMD merely by avoiding asymmetry, represents a respectable yet somewhat one-dimensional perspective. Rather than focusing solely on the elimination of asymmetry, nature-inspired exercises can encourage greater movement variability, bodily awareness, and creative engagement. For example, transferring the gentle swaying of a tree to the violin-playing position or imagining the breath as wind moving clouds while playing the flute can expand musicians' movement repertoire and artistic imagination. Such approaches may help prevent both physical rigidity and habitual patterns of thinking, promoting greater adaptability in performance.

A musician's body is their primary instrument, and it should not be reduced to a mechanical system composed only of anatomical structures that operate the flute or violin. Instead, the body is integrated into its environment; it is a living organism moving through space, enabling musical expression through movements that extend beyond mere instrumental technique.

A limitation of the concept lies in evaluating its outcomes, particularly regarding body-awareness and sound quality. Although subjective physical and musical perceptions are highly relevant for musicians, they can also be misleading: sound quality may be perceived as good even if the performer does not feel well, and vice versa. Moreover, even with blinded expert ratings of recordings, sound quality at a professional level remains largely subjective. Regarding the physical outcomes, the author considers it essential that validated tests should be applied whenever possible. Muscular tension and physical rigidity can be assessed using surface electromyography and motion capture systems. Weight distribution between the feet, resulting from an asymmetrical posture, can be measured with a posturography platform, while pain can be documented through questionnaires or visual analog scales. However, given the multifactorial postural conditions of musicians, improvements in a single variable—for example, pain—do not necessarily indicate that these changes stem from the exercises performed.

Future work should translate the concept into structured educational modules, therapeutic applications, and empirical studies evaluating its effects on posture, movement quality, PRMD, and musical performance. In addition to these practical and research-oriented developments, a broader conceptual shift may be equally important. Expanding one's perspective toward nature's way of adapting to its surroundings and prioritizing function over form can offer musicians a liberating sense of freedom. This broader awareness may help them to play with greater ease, experience less pain, and thrive artistically—even when playing an asymmetrically held instrument.

## Data Availability

The original contributions presented in the study are included in the article/supplementary material, further inquiries can be directed to the corresponding author.
